# NHS England breast screening: Who attends, and why? The influence of psychosocial and demographic determinants

**DOI:** 10.1177/17455057261484363

**Published:** 2026-09-07

**Authors:** Sarah Chapman, Brian Fenech, Haritini Tsangari, Daniel Gaffiero

**Affiliations:** 1 School of Science, Psychology, 151668 University of Derby, Derby, UK; 2 Department of Psychology, Institute of Psychiatry, Psychology and Neuroscience, 4616 King’s College London, London, UK; 3 School of Business, Department of Accounting, Economics and Finance, 121981 University of Nicosia, Nicosia, Cyprus

**Keywords:** breast cancer, breast screening, mammography, barriers, facilitators, health belief model, national health service

## Abstract

**Background:**

Breast cancer is the most common cancer among women in England. However, participation in the NHS England Breast Screening Programme has consistently fallen below the national minimum standard of 70%.

**Objectives:**

The present study aimed to identify psychosocial predictors of breast screening uptake among women aged 50-70.

**Design:**

A cross-sectional questionnaire study was conducted among women aged 50-70 (*n* = 250) living in England.

**Methods:**

The study measured constructs derived from the Extended Health Belief Model, breast cancer awareness, and demographic factors. Binomial logistic regression was used to examine predictors of attendance at participants’ most recent breast screening appointment and intention to attend their next breast screening appointment.

**Results:**

Educational level, perceived benefits of mammography, and perceived barriers to mammography significantly predicted attendance at participants’ most recent breast screening appointment. Intention to attend future screening was predicted by employment status, perceived susceptibility to breast cancer, and beliefs regarding the benefits and barriers of breast screening practices. Perceived benefits and barriers to mammography emerged as the most consistent predictors across both outcomes.

**Conclusion:**

This study highlights key psychosocial and demographic determinants of engagement with the NHS England Breast Screening Programme. Findings suggest that interventions aimed at reducing perceived barriers and increasing awareness of the benefits of breast screening may help improve participation, particularly among underserved groups. Enhancing engagement with breast screening has the potential to support earlier detection and improved breast cancer outcomes.

## Plain language summary

Breast cancer is the most common cancer among women in England. The NHS Breast Screening Programme helps detect breast cancer early, when treatment is more likely to be successful. However, fewer women are attending breast screening than the NHS target recommends. This study explored why some women attend breast screening while others do not. We surveyed 250 women aged 50–70 living in England about their beliefs about breast cancer, their knowledge of breast cancer, and their personal circumstances, such as education and employment. We found that women were more likely to attend breast screening if they believed mammograms were beneficial and if they saw fewer barriers to attending, such as concerns about pain, inconvenience, or fear of the results. Women with higher levels of education were also more likely to have attended their most recent screening appointment. Looking ahead, women who felt they were personally at risk of breast cancer, recognised the benefits of screening, and were employed were more likely to say they intended to attend future appointments. Women with lower levels of education and lower household incomes were less likely to recognise important signs and symptoms of breast cancer, suggesting that improving breast cancer awareness could help reduce inequalities. These findings suggest that increasing breast screening attendance will require more than simply encouraging women to attend. Practical improvements, such as easier appointment booking, reminder systems, and clear, accessible information about the benefits of screening and common concerns, may help more women participate in the NHS Breast Screening Programme and support earlier detection of breast cancer.

## 1. Introduction

Breast cancer (BC) is the most common cancer among women in England, with the incidence rate rising over the last decade, and approximately one in seven women diagnosed during their lifetime.^1,2^In England, 2022 saw 50,633 cases, an increase from 45,790 in 2017.^3,4^ Approximately 80% of cases occur in women over 50, with BC risk increasing with age.^5^ The physical and psychological impact of BC is significant, with women experiencing fear of recurrence, negative body image, and long-term depression and anxiety.^6–8^ Therefore, diagnosing BC early is essential to reduce the likelihood of more radical treatment, limit impact on quality of life and improve survival rates.^9–11^ The NHS England Breast Screening Programme (NHS England BSP), introduced in 1988, invites women aged 50-70 for screening every three years, with the aim of detecting BC at an early stage to optimise health outcomes.^12,13^

Despite the benefits of screening, participation remains suboptimal. Women automatically receive their first invite between the ages of 50-53; however, 46.3% fail to respond to their initial invitation.^14,15^ The NHS England BSP prevents around 1300 deaths annually, yet participation has fallen below the national minimum standard of 70%, with only 64.6% of nearly three million eligible women attending in 2022-23.^15,16^ In 2022/23, the NHS England BSP detected 18,942 BC cases, and projections suggest that increasing participation could substantially increase cancer detection rates.^15,17^

Research exploring factors influencing participation within the NHS England BSP remains relatively sparse. Existing studies have focused on broader UK populations,^18^ other countries,^19,20^ specific minority groups,^21^ or particular regions of England.^22^ Furthermore, several studies rely primarily on qualitative methods, limiting the generalisability and applicability of findings to wider policy and practice.^23^ This evidence gap limits the NHS’s ability to develop targeted, evidence-based interventions to address declining participation.

Several theoretical frameworks have been applied to understand screening behaviours, including the Theory of Planned Behaviour^24^ and the COM-B Model.^25^ However, whilst successfully applied to cancer screening, these frameworks may not adequately capture factors specific to breast screening behaviour.^26–28^ The Extended Health Belief Model (EHBM),^29^ has been widely used to explain psychological and behavioural determinants of breast screening uptake. Women who perceive themselves to be susceptible to breast cancer and understand its potential severity are more likely to participate in screening.^30,31^ Greater perceived benefits, self-efficacy, and cues to action, may facilitate participation,^32^ whereas perceived barriers such as embarrassment, fear of BC diagnosis and anticipated pain may reduce uptake.^33–35^ Health motivation and sociodemographic factors have also been associated with screening engagement.^36–38^

The present study aimed to examine psychological, behavioural and sociodemographic predictors of breast screening participation among women aged 50-70 eligible for the NHS England BSP. Specifically, the study examined whether EHBM constructs, breast cancer awareness, and sociodemographic characteristics predicted attendance at participants’ most recent NHS England BSP appointment and intention to attend their next scheduled breast screening appointment. It was hypothesised that these factors would significantly predict both previous screening attendance (Hypothesis 1) and future screening intention (Hypothesis 2).

## 2. Methods

### 2.1. Study design

The present study employed a cross-sectional questionnaire design comprising 16 predictor variables, including constructs from Champion’s CHBMS (6),^39^ breast cancer awareness (1) and demographic factors (9). The predictors were perceived susceptibility, severity, benefits, barriers, self-efficacy, health motivation, breast cancer awareness, age, nationality, ethnicity, marital status, religion, educational level, employment status, household income and family history of breast cancer. The outcome variables were dichotomous: attendance of participants’ most recent breast screening appointment (yes vs. no) and intention to attend their next scheduled breast screening appointment (yes vs. no), as part of the NHS England Breast Screening Programme. The reporting of this study conforms to the STROBE statement.^40^

### 2.2. Participants

Initially, a total of 370 female participants living in England were recruited through online advertisements distributed via online platforms, including Breast Cancer Now and Against Breast Cancer charities, and social media sites including X, Reddit, Facebook and LinkedIn. Advertisements contained a brief description of the study and a link to the online Qualtrics questionnaire, allowing interested individuals to self-select into participation. Participant recruitment, eligibility assessment, exclusions, and inclusion in the final analytical sample are summarised in Figure 1 below. The NHS England BSP invites women aged 50-70, so the present study focused on this age group.

**Figure 1.**
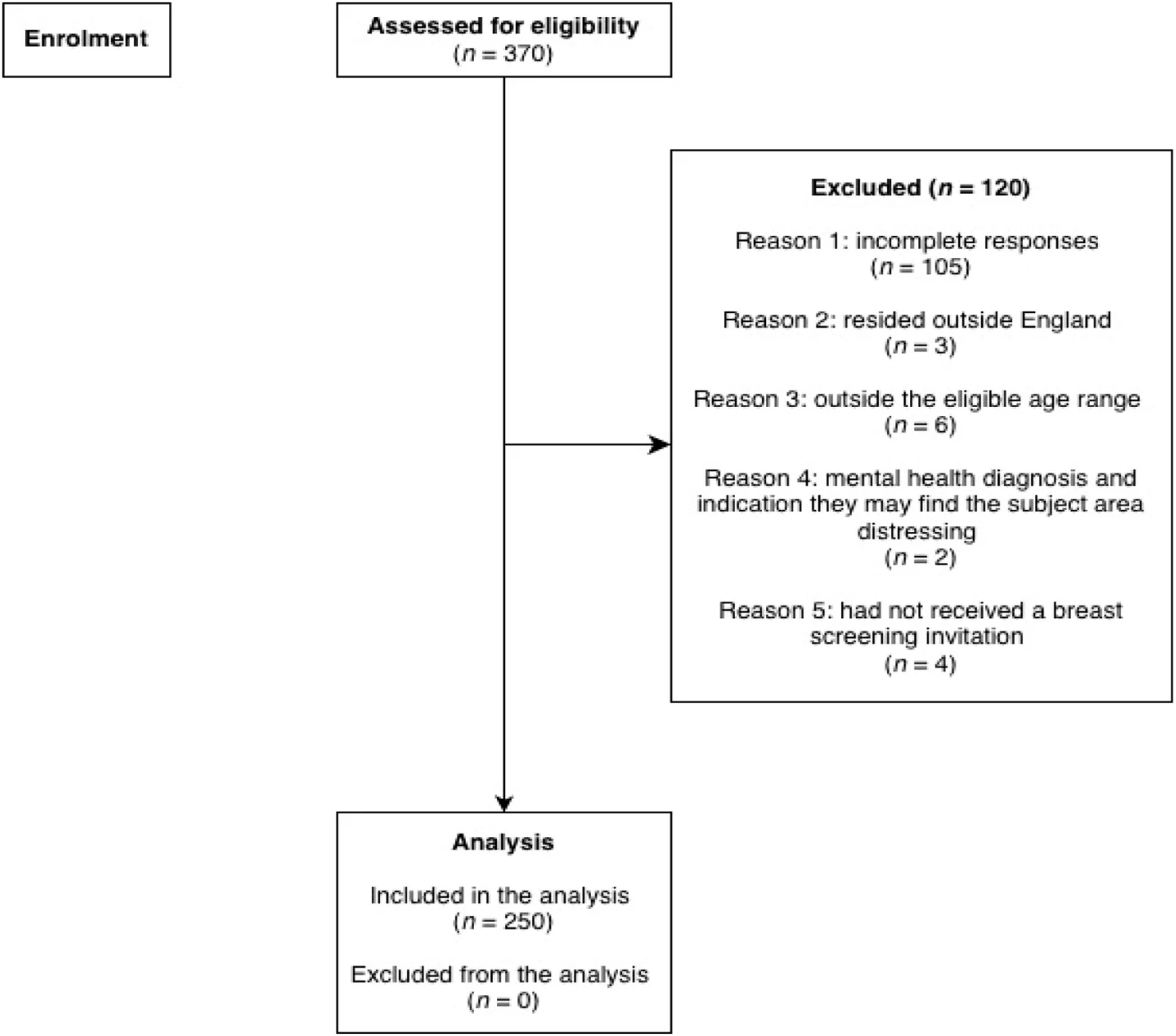
Participant recruitment, eligibility assessment, exclusions and final analytical sample. *Note.* ‘N’ = Number of participants.

Of those recruited, 105 respondents were excluded due to incomplete responses, three resided outside England, six were outside the eligible age range, two had a mental health diagnosis and indicated they may find the subject area distressing, and four had not received a breast screening invitation. Incomplete responses were handled using listwise deletion, and no data imputation procedures were undertaken.

A small number of participants (*n* = 28) reported that they had not received an invitation within the previous three years but had attended their most recent screening appointment. These participants were retained because the primary outcome reflected attendance behaviour rather than strict adherence to invitation timing. Following application of the eligibility criteria and removal of incomplete responses, the final sample comprised 250 participants (response rate 67.57%; age range, 50-70 years old; mean ± SD, 58.25±5.39). Demographic data are presented in Table 1.

**Table 1.** Sociodemographic characteristics of study participants (N=250).

Demographic characteristics	n	%
**Age range**		
50-55	92	37.0
56-60	67	27.0
61-65	62	25.0
66-70	29	11.0
**Ethnicity**		
White	239	95.6
Mixed	3	1.2
Asian	4	1.6
Black	2	0.8
Other	2	0.8
**Nationality**		
British	241	96.0
Other	9	4.0
**Religion**		
Christianity	140	56.0
Hinduism	1	0.4
Judaism	16	6.0
Sikhism	1	0.4
Other	7	3.0
No religion	82	33.0
Prefer not to say	3	1.2
**Marital Status**		
Married/Civil Partner	165	66.0	​
Single	31	12.0
Divorced/Widowed	39	16.0
Other	15	6.0
**Educational Level**		
Primary Education	2	0.8
Secondary School	79	31.6
Bachelor’s degree	92	36.8
Postgraduate degree	46	18.4
Other	29	11.6
No formal education	2	0.8
**Employment Status**		
Employed	176	70.4
Unemployed	12	4.8
Student	1	0.4
Other	61	24.4
**Annual Household Income**		
Less than £10,000	6	2.4
£10,001- £20,000	23	9.2
£20,001- £30,000	43	17.2
£30,001- £40,000	45	18.0
£40,000 +	133	53.2
**Invitation to Breast Screening in last 3 years**		
Yes	222	88.8
No	28	11.2
**Family History of Breast Cancer**		
Yes	81	32.0
No	162	65.0
Unsure/Don’t know	7	3.0

*Note.* ‘N’ = Number of participants.

A prospective power analysis using G*Power indicated that a sample size of 190 would be sufficient to detect a medium effect size with 80% power at *α* = .05. Eligibility criteria included: i) Women aged 50-70, ii) residency in England; and iii) prior invitation to breast screening through the NHS England BSP. No financial incentives were offered; however, University of Derby participants received course credit.

### 2.3. Materials

#### 2.3.1. Demographic questionnaire

Participants provided information on their age, nationality, ethnicity, marital status, religion, educational level, employment status, household income and family history of BC. They also answered four questions about the NHS England BSP, including whether they had been invited to participate in breast screening in the past three years (i), attended their last breast screening appointment (ii), intended to attend their next breast screening appointment (iii), and their reasons for past or anticipated non-attendance (iv). The full questionnaire is available in Appendix A.

#### 2.3.2. Champion’s health belief model scale (CHBMS)

To measure the constructs of the EHBM in relation to breast screening (mammography) and breast self-examination, the present study utilised Champion’s (1999) scale.^39^ The scale consists of 53 items, across eight subscales: Perceived susceptibility (5), seriousness (perceived severity) (7), benefits of breast self-examination (6), barriers to breast-self-examination (6), self-efficacy (11), health motivation (7), benefits of mammography (6) and barriers of mammography (5). Responses were measured on a 5-point Likert Scale ranging from 1 = ‘*Strongly disagree’* to 5 = ‘*Strongly agree’*. Total scores were calculated for each construct, with higher scores reflecting greater perceived susceptibility, severity, benefits, barriers, self-efficacy and health motivation. Cronbach’s α for the subscales is reported between .61 and .93, indicating good to excellent internal consistency and a high degree of reliability in the measurement of the constructs of Champion’s CHBMS (Champion, 1999).

Permission to use and modify the scale was granted by Champion (1999; Appendix B). US-specific terms were adapted to UK equivalents (e.g., “*10 pence*” instead of “*quarter*” and “*5 pence*” instead of “*dime*”). Additionally, one question - “*Having a mammogram or x-ray of the breast would cost too much money*” - was removed as it was irrelevant in the UK context, where the NHS England BSP is free. The full scale and modifications are provided in Appendix B.

#### 2.3.3. Breast cancer awareness measure (BCAM)

Participants’ knowledge and awareness of BC were assessed using the Breast Cancer Awareness Measure (BCAM; Linsell et al., 2010).^41^ Whilst Champion’s CHBMS measures barriers to mammography, such as lack of BC awareness, the BCAM can assess specific gaps in BC knowledge, for example awareness of BC risk factors and recognition of non-lump BC symptoms.^42^ By complementing the EHBM and Champion’s CHBMS, the BCAM identifies key areas of low BC awareness, and contributes to informing targeted strategies, such as educational interventions, to increase health literacy. The BCAM consists of three questions. The first measures awareness of BC warning signs. Participants were presented with 11 possible warning signs, including nine non-lump and two lump-related symptoms (e.g., changes in the shape of your breast or nipple, or a lump or thickening in your breast) and asked to select those they recognised. To be classified as aware, participants needed to correctly identify at least five out of the nine non-lump warning signs. The second assesses awareness of age-related risk, here participants were asked to choose one of four options to indicate which age group is most susceptible to BC. To demonstrate awareness, they needed to correctly identify that a “*70-year-old woman*” is most likely to develop BC within the next year. The third evaluates awareness of breast self-examination. Participants were asked to indicate the frequency with which they check their breasts by selecting one of four response options. To be considered aware, they needed to report performing self-examination at least once a week or once a month.

Participants who answered all three questions correctly were classified as ‘breast cancer aware’. This BCAM is reported as a valid and reliable measure, with good construct validity. The full scale is provided in Appendix C.

#### 2.3.4. Outcome measure: Breast screening uptake

To assess participants’ engagement with breast screening as part of the NHS England BSP, they were asked whether they had attended their most recent breast screening appointment (yes or no), and whether they intended to attend their next scheduled breast screening appointment (yes or no). Participants who had not attended or did not plan to attend were also asked to provide their reasons for non-attendance.

### 2.4. Procedure

The online survey was developed using Qualtrics XM software (Provo, UT; version January to April 2025) and was conducted between 16^th^ January 2025 and 14^th^ April 2025. The present study adhered to the British Psychological Society Code of Human Research Ethics (2021) and the BPS Ethics guidelines for internet mediated research (2021). Ethical approval was obtained from the University of Derby College of Health, Psychological and Social Care Research Ethics Committee (approval no. *ETH2425-1565*). All participants provided informed written consent before taking part in the study. They first completed the demographic questionnaire, followed by Champion’s CHBMS, the BCAM, and the NHS England BSP questionnaire. On average, participants took 37 minutes to complete the survey.

### 2.5. Statistical analysis

Statistical analyses were conducted using SPSS (version 26; IBM Corp.). To examine the predictors of engagement with breast screening uptake among women aged 50-70 participating in the NHS England BSP, two binomial logistic regression analyses were performed. The predictor variables included demographic factors, Champion’s CHBMS constructs, and breast cancer awareness. The two outcome variables were i) attendance of the most recent appointment (yes vs. no) and ii) intention to attend the next scheduled appointment (yes vs. no) as part of the NHS England BSP.

Due to small sample sizes within some categories, several demographic variables were recoded prior to analysis. Marital status was recoded as married vs. not married; household income as low/middle (<£40,000) vs high (£40,000+); educational level collapsed into lower education (no formal education, primary or secondary education, and other) and higher education (bachelor’s or postgraduate degree), and employment status collapsed into employed vs. unemployed, to ensure adequate cell sizes for logistic regression.

## 3. Results

### 3.1. Data screening

To ensure the robustness of the analyses, data were screened for outliers, multicollinearity and linearity. Potential outliers, identified by Z-scores (*n* = 14), were observed for severity/seriousness, benefits (BSE), barriers (BSE), self-efficacy, benefits (mammogram) and barriers (mammogram). Upon further inspection, these values were within the plausible range for the respective scales and were considered valid responses; therefore, no cases were excluded from the analysis. Multicollinearity diagnostics indicated no concerns, with all tolerance values exceeding .10, suggesting acceptable values of intercorrelation among the predictor variables. The assumption of linearity between continuous predictors and the logit of the outcomes (i.e., most recent breast screening appointment and whether they intended to attend their next scheduled breast screening appointment) was assessed using the Box–Tidwell procedure, and no violations were detected.

To test the hypotheses that perceived susceptibility, severity, benefits, barriers, self-efficacy, health motivation, BC awareness, and sociodemographic variables would significantly predict (a) attendance of the most recent NHS England breast screening appointment, and (b) intention to attend the next scheduled breast screening appointment, two binomial logistic regression analyses were conducted. Given most respondents were British (96.4%) and of white ethnicity (95.6%), nationality and ethnicity were not included in the regression model.

### 3.2. Inferential statistics

#### 3.2.1. Examining the predictors of attendance of the most recent breast screening appointment

Religion was excluded due to issues with model stability. Following model refinement, age, marital status, employment status, income level, BC awareness and BC history were removed because they did not significantly contribute to the model (all *p* > .05). The final model included educational level and EHBM constructs. Model fit statistics are reported in Supplementary Table S1.

The final model identified educational level, perceived benefits of mammography and perceived barriers to mammography as significant predictors of attendance at the most recent breast screening appointment (Table 2). Women with higher educational attainment (i.e., bachelor’s degree or equivalent/postgraduate qualification) were approximately 4.8 times more likely to have attended their most recent breast screening appointment than women with lower or secondary education (OR = 4.76, 95% CI [1.24, 18.29], *p* = .023). However, the CI was wide and should therefore be interpreted with caution.

**Table 2.** Predictors of attendance at most recent breast screening appointment.

​	B	S.E.	Wald	df	Sig.	Exp(B)	95% CI for Exp(B)	
							Lower	Upper
**Educational level**	**1.560**	**.687**	**5.152**	**1**	**.023** ^**^	**4.757**	**1.237**	**18.287**
Susceptibility	.170	.094	3.282	1	.070	1.186	.986	1.425
Seriousness	.008	.084	.008	1	.928	1.008	.854	1.189
Benefits (BSE)	.015	.111	.018	1	.895	1.015	.816	1.262
Barriers (BSE)	.128	.115	1.229	1	.268	1.136	.906	1.425
Self-efficacy	-.008	.051	.025	1	.873	.992	.897	1.096
Health Motivation	.092	.081	1.284	1	.257	1.096	.935	1.284
**Benefits (Mammogram)**	**.331**	**.099**	**11.191**	**1**	**<.001** ^*^	**1.393**	**1.147**	**1.691**
**Barriers (Mammogram)**	**-.319**	**.124**	**6.653**	**1**	**.010** ^*^	**.727**	**.570**	**.926**

B, unstandardised regression coefficient; S.E., standard error of the B coefficient; Wald, Wald statistic; df, degrees of freedom; Sig., significance level; Exp(B), odds ratio; 95% CI for Exp(B), 95% confidence interval for the odds ratio; *P < .01, **P < .05.

Perceived benefits of mammography were positively associated with attendance, with each one-unit increase corresponding to a 39% increase in the odds of attendance (OR = 1.39, 95% CI [1.15, 1.69], *p < .001*). Conversely, perceived barriers to mammography were negatively associated with attendance, with each one-unit increase corresponding to a 27% reduction in the odds of attendance (OR = .73, 95% CI [.57, .93], *p* = .010). No other predictors significantly contributed to the model (all *p* > .05).

#### 3.2.2. Examining the predictors of intention to attend the next scheduled breast screening appointment

A second binomial logistic regression analysis was conducted to examine predictors of women’s intention to attend the next scheduled breast screening appointment. Religion was excluded due to model instability. Following model refinement, age, marital status, educational level, income level, BC awareness and BC history were removed because they did not significantly contribute to the model (*p* > .05). The final model included employment status and EHBM constructs. Model fit statistics are also reported in Supplementary Table S1.

The final model identified employment status, perceived susceptibility, perceived benefits of breast self-examination (BSE), perceived benefits of mammography and perceived barriers to mammography as significant predictors of intention to attend the next scheduled breast screening appointment (see Table 3 below). Women who were employed were significantly more likely to report intention to attend their next breast screening appointment than women who were unemployed (OR = 53.34, 95% CI [3.79, 751.57], *p* = .003). However, the wide CI indicates considerable uncertainty regarding the magnitude of the association.

**Table 3.** Predictors of intention to attend next scheduled breast screening appointment.

​	B	S.E.	Wald	df	Sig.	Exp(B)	95% CI for Exp(B)	
							Lower	Upper
**Employment status**	**3.977**	**1.350**	**8.680**	**1**	**.003** ^*^	**53.338**	**3.785**	**751.565**
**Susceptibility**	**.434**	**.159**	**7.472**	**1**	**.006** ^*^	**1.543**	**1.131**	**2.107**
Severity (Seriousness)	-.073	.101	.524	1	.469	.930	.763	1.133
**Benefits (BSE)**	**.377**	**.166**	**5.165**	**1**	**.023** ^**^	**1.458**	**1.053**	**2.019**
Barriers (BSE)	.127	.135	.889	1	.346	1.135	.872	1.478
Self-efficacy	-.030	.060	.250	1	.617	.971	.863	1.091
Health Motivation	-.221	.116	3.618	1	.057	.802	.638	1.007
**Benefits (Mammogram)**	**.494**	**.150**	**10.902**	**1**	**< .001** ^*^	**1.639**	**1.222**	**2.198**
**Barriers (Mammogram)**	**-.419**	**.186**	**5.079**	**1**	**.024** ^**^	**.658**	**.457**	**.947**

B, unstandardised regression coefficient; S.E., standard error of the B coefficient; Wald, Wald statistic; df, degrees of freedom; Sig., significance level; Exp(B), odds ratio; 95% CI for Exp(B), 95% confidence interval for the odds ratio; *P < .01, **P < .05.

Greater perceived susceptibility to breast cancer was associated with a higher likelihood of intending to attend future screening, with each one-unit increase corresponding to a 54% increase in the odds of attendance intention (OR = 1.54, 95% CI [1.13, 2.11], *p* = .006).

Perceived benefits of BSE were also positively associated with attendance intention, with each one-unit increase corresponding to a 46% increase in the odds of intending to attend future screening (OR = 1.46, 95% CI [1.05, 2.02], *p* = .023). Similarly, perceived benefits of mammography were associated with a 64% increase in the odds of attendance intention (OR = 1.64, 95% CI [1.22, 2.20], *p <* .001). Conversely, perceived barriers to mammography were associated with a reduced attendance intention, with each one-unit increase corresponding to a 34% reduction in the odds of intending to attend future screening (OR = .66, 95% CI [.46, .95], *p* = .024). All remaining predictors were non-significant (all *p* > .05).

#### 3.2.3. Exploring the association between demographic variable predictors and questions of the BCAM

A Chi-Square test for association was conducted between demographic variable predictors (marital status, employment status, education level, income level and BC history) and all three questions of the BCAM (correct vs. incorrect). The first question (Q1) asked participants to correctly identify at least five out of the nine listed non-lump warning signs. The second question (Q2) assessed awareness of age-related risk, with participants asked to correctly identify from a choice of four options that a “*70-year-old woman*” is most likely to develop BC within the next year. The third question (Q3) asked participants to indicate from a choice of four options the frequency with which they check their breasts and correctly identify “*at least once a week or once a month*”. Expected cell frequencies were all >5. There was a statistically significant association between education and Q1, with significantly more women with a higher-level education (i.e., bachelor’s or equivalent/postgraduate degree) correctly identifying more than five non-lump BC symptoms than women with low/secondary education (84.2 vs. 62.4%), χ^2^ (1, *N* = 250) = 15.086, *p* < .001. There was a small-to-moderate association (φ = -.246) between education level and question one. Frequencies are reported in Table 4 below.

**Table 4.** Associations between sociodemographic variables and breast cancer awareness.

​	Q1.* Correct (%)	Q1.* Incorrect (%)	Total
Higher education	139 (84.2)	26 (15.8)	165
Low/secondary education	53 (62.4)	32 (37.6)	85
Total	192 (76.8)	58 (23.2)	250
High income	111 (83.5)	22 (16.5)	133
Low/middle income	81 (69.2)	36 (30.8)	117
Total	192 (76.8)	58 (23.2)	250

​	Q2.* Correct (%)	Q2.*Incorrect (%)	Total
Higher education	21 (12.7)	144 (87.3)	165
Low/secondary education	2 (2.4)	83 (97.6)	85
Total	23 (9.2)	227 (90.8)	250

​	Q3.* Correct (%)	Q3.^*^ Incorrect (%)	Total
Employed	109 (66.9)	54 (33.1)	163
Not employed	43 (49.4)	44 (50.6)	87
Total	152 (60.8)	98 (39.2)	250

*Q1. Identify 5 or more non-lump symptoms of BC.

**Q2^.^ In the next year, who is more likely to get BC? (Correct answer: a 70-year-old woman).

***Q3. How often do you check your breasts? (Correct answer: at least once a week/month).

There was a statistically significant association between income level and Q1, with significantly more women with a high income correctly identifying more than five non-lump BC symptoms than women with low/middle income (83.5%, vs. 69.2%), χ^2^ (1, N = 250) = 7.072, *p* = .008, φ = -.168, indicating a small association. There was also a statistically significant association between education level and Q2. Knowledge of age-related BC risk was low overall (9.2% correct), however significantly more women with a higher-level education correctly identified that a 70-year-old woman is most likely to develop BC in the next year than women with low/secondary level education (12.7% vs. 2.4%), χ^2^ (1, N = 250) = 7.228, *p* = .007, φ = -.170, indicating a small association.

Finally, there was a statistically significant association between employment status and Q3, with significantly more employed women correctly indicating they checked their breasts at least once a week/month than unemployed women (66.9% vs. 49.4%), χ^2^ (1, N = 250) = 7.244, *p* = .007, φ = .170, indicating a small association. All other associations were non-significant (*p* > .05).

#### 3.2.4. Exploring participant reasons for non-attendance of breast screening appointments

A total of 18 participants provided a reason they selected ‘*no*’ for non-attendance of their most recent breast screening appointment and ‘*no*’ for intention to attend their next scheduled breast screening appointment, as summarised in Table 5. Although the sample size in Table 5 is small, the results do provide some additional insight, in terms of the reasons for non-attendance at breast screening appointments.

**Table 5.** Participant-reported reasons for non-attendance at recent and future breast screening appointments.

Category	Reason	Previous appointment (N=14)	Next appointment (N=10)
Barriers (6)	Forgot/put off making appointment	5	-
	Fear of mammography pain	-	1
Medical (6)	Under 5-year pathway following BC treatment	2	2
	Receiving regular Cancer CT scans	2	1
	Medical PTSD	1	1
	Previous mastectomy	-	1
Lack of BC Awareness (1)	BC over medicalised (self-resolving)	1	-
	Absence of BC symptoms	-	1
Safety Concerns (2)	Ultrasound is safer than mammogram	1	2
Susceptibility (1)	Low risk of BC (research study participation)	1	-
Eligibility (2)	Already attended the previous year	1	**-**
	Will be outside eligible age range	-	1

Note: Numbers in parentheses indicate the number of participants in each category.

BC = Breast Cancer.

## 4. Discussion

The present study investigated predictors of participation in the NHS England Breast Screening Programme among women aged 50-70, including constructs from the Extended Health Belief Model, breast cancer awareness measure and demographic factors. Participation in the NHS England BSP has consistently fallen below the national minimum standard of 70%,^15,16^ highlighting the need to better understand the factors influencing participation. This is critical for informing strategies to increase engagement with the programme and reduce BC mortality through early detection. The results partially support hypothesis 1, revealing educational level, perceived benefits and perceived barriers of mammography as significant predictors of most recent breast screening appointment attendance. Hypothesis 2 was also partially supported, with employment status, perceived susceptibility to BC, perceived benefits of BSE, perceived benefits and perceived barriers of mammography emerging as significant predictors of intention to attend the next scheduled breast screening appointment.

Taken together, these findings highlight the importance of psychosocial and behavioural determinants of breast screening attendance. Consistent with the EHBM and previous research, women who perceived greater barriers to mammography, such as fear of BC diagnosis, mammography pain, and prioritisation of other factors (e.g., work or family),^43–45^ were significantly less likely to have attended their most recent appointment or intend to attend future appointments.^46,47^ Lower levels of educational attainment significantly predicted non-attendance, suggesting that lower educational attainment may be associated with reduced health literacy, potentially contributing to perceived barriers such as fear and uncertainty regarding breast screening. International evidence from Cyprus, Hungary, Turkey and the US supports this, indicating that college degrees or higher facilitate both breast screening attendance and breast self-examination (BSE); higher levels of education may reflect greater health literacy and understanding of preventive health measures.^48–51^ Interestingly, several EHBM constructs, including perceived severity, self-efficacy and health motivation, were not significant predictors after accounting for other variables. This may suggest that beliefs relating specifically to the perceived benefits and barriers of mammography are more influential in determining screening participation than broader health beliefs within this population.

The present study identified key socio-economic barriers to breast screening uptake, with low educational attainment and unemployment identified as significant predictors of non-attendance. Additionally, lower income and education levels were associated with limited breast cancer awareness. These findings align with international research, highlighting both individual and structural barriers to breast screening which are consistent across global healthcare systems.

Evidence shows that unemployment remains a common barrier to breast screening attendance internationally, despite offering publicly funded breast screening programmes. Research conducted in South Africa suggests that this may be attributable to childcare responsibilities limiting women’s ability to work, resulting in family obligations being prioritised over their own health.^52^ A Chilean study identified that unemployed women were more likely to experience marginalisation in accessing healthcare, which may contribute to lower mammogram attendance.^53^ Conversely, women in employment in Finland experienced fewer barriers to breast screening, as higher income may facilitate access through both private healthcare and the national screening programme.^54^ This international evidence highlights the persistent international socioeconomic inequalities that women face in accessing and participating in preventive healthcare settings. In France, where both organised and opportunistic breast screening is available, unemployed women are less likely to attend opportunistic breast screening, due to experiencing exclusion arising from out-of-pocket costs reimbursable only through private health insurance.^55^ In countries such as Brazil, where breast screening is available solely through opportunistic delivery, research has highlighted that socioeconomic inequalities such as unemployment and lower educational attainment persist in negatively influencing participation.^56^ Consistent with the present study, difficulties women reported regarding booking appointments, emphasise the need to address barriers to breast screening access at both individual and systemic levels.

In the present study, perceived benefits of both BSE and mammography, and perceived susceptibility was associated with attendance, consistent with prior research.^30,32,57^ Supplementary analyses suggested that some participants perceived ultrasound to be a safer screening modality than mammography. These findings suggest that communicating reassurance that mammography remains the gold standard of BC diagnosis,^58^ and that any risks, for example overdiagnosis, are low and far outweighed by benefits. Emphasising health advantages, such as the likelihood of successful treatment outcomes for early-stage BC detection, may help address barriers to participation by combatting misinformation, and encouraging positive attitudes towards breast screening.^59–61^

Supplementary analyses also revealed that forgetting to book or attend appointments and medical issues were the most frequently cited participant reasons for non-attendance. The current NHS booking system lacks consistency by issuing either booking invitations or pre-booked appointments. Given these findings and considering that 46.3% of women fail to respond to their first screening invitation,^15,62^ placing the responsibility on women to book their own appointment may constitute an unnecessary barrier to participation. That said, trials of a new ‘ping and book’ initiative to digitise the mammogram booking system are underway, which include issuing mobile phone alerts to book via the NHS app with follow-up email, text and postal reminders.^63^ Whilst research indicates appointment reminders considerably improve screening participation,^32^ the findings of the present study suggest adopting a pre-booked system with follow-up prompts may promote inclusivity, reduce burden on women and mitigate perceived barriers.

Additionally, being under a five year pathway following BC or receiving regular Computed Tomography (CT) scans were cited as reasons for non-attendance of breast screening, aligning with previous research which suggests women with comorbidities such as cancer are less likely to engage in breast screening participation.^64^ This is potentially due to conflicting health priorities, highlighting the need to facilitate uptake among this group by removing perceived physical and psychological barriers to screening participation.

Although BC awareness did not emerge as a significant independent predictor in either regression model and was therefore excluded from the final regression analyses, supplementary analyses identified important associations between breast cancer awareness and sociodemographic factors. Specifically, significant gaps in awareness of non-lump breast cancer symptoms were observed among women with lower educational attainment and household incomes of ≤ £40,000, both of which have previously been associated with lower screening participation.^42^ These findings suggest that breast cancer awareness may not directly predict screening behaviour once psychological and demographic variables are considered simultaneously. Nevertheless, they may represent an important contextual influence associated with groups at increased risk of lower screening engagement. Consistent with prior research, participants’ reasons for non-attendance also suggested that lack of awareness may contribute to screening disengagement through unfamiliarity with BC symptoms and the benefits of mammography,^41,65^ including beliefs that BC may self-resolve, or that mammography is only necessary when symptoms are present.

The present study also found that perceived susceptibility to BC emerged as a significant predictor of intention to attend the next scheduled breast screening appointment. This is consistent with the EHBM and previous research which indicates that personal or family history of BC increases women’s perceptions of susceptibility to BC risk, facilitating breast screening participation.^30,57^ Whilst BC history was not a significant predictor of screening participation, findings align with previous research suggesting that direct experience of previous breast issues is sufficient to increase perceived susceptibility to BC and therefore facilitate breast screening participation.^66^ Supplementary analyses revealed low perceived risk of BC as a reason for non-attendance of previous breast screening and combined with the present study’s findings on lack of BC risk awareness, suggest that women may have excluded risk factors pertinent to them into assessment of their own susceptibility. Communicating relevant breast cancer risk factors to women eligible for screening, alongside strategies to reduce modifiable risk where appropriate, may improve perceived susceptibility and promote screening uptake.

## 5. Limitations and future research

The present study has certain limitations. Firstly, the sample consisted of predominantly white British participants, limiting the generalisability of the findings to other populations. The association between lower mammogram participation and ethnic minorities has been well documented in research across several countries,^67–69^ and considerable barriers remain, notably lack of BC knowledge, language, religion and cultural beliefs. Within ethnic minority groups, barriers are most prevalent amongst less-educated women and lower socio-economic status, however, there remains a paucity of research within the English breast screening context.^18,21,68^ The present study excluded religion as a predictor due to model instability. Future research should therefore seek to recruit more ethnically, culturally and religiously diverse samples to improve the representativeness and applicability of findings.

Secondly, participants were recruited through online platforms and social media, which may have introduced selection bias. Individuals who engage with online health-related content may be more digitally literate, health-conscious, or motivated to participate in preventive health behaviours than the wider population. As such, the findings may not reflect the experiences and beliefs of women who have limited internet access or lower levels of digital engagement. Future studies should consider combining online recruitment with community-based or healthcare-based recruitment approaches to improve sample representativeness.

Thirdly, all variables were assessed using self-report measures. Thus, responses may have been influenced by recall bias or social desirability bias. That said, self-report measures are widely used in health psychology research; future studies may benefit from incorporating objective screening records where possible.

Fourthly, confidence intervals were wide for some predictors (e.g., educational level and employment status) and therefore should be interpreted with caution. Additionally, a substantial proportion of respondents were excluded due to incomplete survey responses. Although analyses were conducted using completed cases only, it is possible that participants who exited the survey differed from those who fully completed it.

Finally, the data included in the binomial logistic regression analyses were highly skewed towards women reporting attendance at their most recent breast screening appointment (‘*yes*’, n = 235; ‘*no*’, n = 15) and intention to attend future screening (‘*yes*’, n = 239; ‘*no*’, n = 11). This imbalance may have contributed to higher sensitivity and lower specificity within the models. Future research should utilise targeted recruitment strategies to reach women who are hesitant about, or disengaged from, breast screening programmes.

## 6. Conclusion

The present study identified psychosocial and demographic factors influencing participation in the NHS England Breast Screening Programme among women aged 50-70 years. Employment status, educational level, perceived susceptibility to BC, and perceived benefits of mammography and BSE were associated with greater screening engagement, whereas perceived barriers to mammography were associated with lower participation. Importantly, the findings suggest that barriers to screening extend beyond general negative attitudes, and include practical obstacles such as forgetting appointments, delayed booking, competing priorities or health-related concerns. Furthermore, lower awareness of breast cancer symptoms and risk factors was associated with poorer performance on measures of breast cancer awareness, particularly among women with lower educational attainment and household income. These findings indicate that interventions should move beyond simply encouraging screening attendance and instead target specific barriers through tailored reminders, accessible appointment systems, culturally appropriate educational materials, and initiatives designed to improve breast cancer literacy among underserved populations.

## Supplemental material

Supplemental material - NHS England breast screening: Who attends, and why? The influence of psychosocial and demographic determinants Supplemental material for NHS England breast screening: Who attends, and why? The influence of psychosocial and demographic determinants by Sarah Chapman, Brian Fenech, Haritini Tsangari and Daniel Gaffiero in Women's Health.

## Data Availability

Data and materials are available upon reasonable request.*
